# Interrupting sedentary behaviour when working from home: a qualitative exploration of older desk-based employees

**DOI:** 10.1186/s12889-026-26719-4

**Published:** 2026-02-19

**Authors:** Lily Mott, Amelia Parchment, Annemarie Money, Sheena Johnson, Chris Todd

**Affiliations:** 1 Faculty of Biology, Medicine & Health, School of Health Sciences, The University of Manchester, Manchester, M13 9PL UK; 2 NIHR Applied Research Collaboration - Greater Manchester, School of Health Sciences, The University of Manchester, Manchester, M13 9PL UK; 3 Manchester Academic Health Science Centre, Manchester, M13 9NQ UK; 4 Alliance Manchester Business School, The University of Manchester, Manchester, M15 6PB UK; 5 Healthy Ageing Research Group, Jean Macfarlane Building, The University of Manchester, Manchester, M13 9PY UK

**Keywords:** Sedentary behaviour, Working from home, Desk-based workers, Behaviour change wheel, Intervention

## Abstract

Desk-based employees sit for large amounts of their day, exposing them to the health risks associated with sedentary behaviour (SB). The number of older workers (≥ 50) is increasing, making it important to promote healthy ageing in the workplace. With more workplaces offering home-working, it is important to explore how this environment influences SB. This study uses the Theoretical Domains Framework (TDF) alongside the Capability, Opportunity, Motivation-Behaviour (COM-B) model. Twenty-two interviews were conducted and inductive reflexive thematic analysis is used to develop six themes before deductively mapping these to the TDF and COM-B model: *(1) Ambiguous knowledge of sedentary behaviour (2) Nature and demand of professional role in conflict with behaviour change (3) Lack of conscious action in relation to sedentary behaviour (4) Health as potential motivator for older employees (5) There is hope for targeting workplace sitting at home* and *(6) Interrupting sedentary behaviour not actively embedded in homeworking culture.* SB is influenced by numerous complex factors. The blend of conducting work tasks at home plays an important role in SB and future work should consider how interventions can be implemented across homeworking environments. The experience and needs of older employees should be considered and used to target motivation.

**Supplementary Information:**

The online version contains supplementary material available at 10.1186/s12889-026-26719-4.

## Background

Sedentary behaviour (SB) is defined as ‘any waking behaviour characterized by an energy expenditure ≤ 1.5 metabolic equivalents (METs) while in a sitting, reclining, or lying posture’ [1]. High levels and prolonged bouts of SB have been associated with increased risk for all-cause mortality, type 2 diabetes, some cancers, cardiovascular disease, metabolic syndrome, anxiety, and depression [2, 3]. Some evidence suggests that interrupting prolonged bouts of SB with short bouts of light to moderate physical activity (PA) can yield positive effects on cardiometabolic parameters, which may be more beneficial than interrupting sitting with standing [4].

Desk-based workers spend large amounts (between 58% and 82%) of their working day seated, often in uninterrupted bouts [5–9]. This exposes them to the adverse health implications associated with SB, including increased risk of cardiovascular disease, type 2 diabetes, and all-cause mortality [10, 11]. Existing interventions for office-based employees often target one or more of the complex and interacting elements that influence SB, including individual, environmental, political, and social factors [12]. There exists a large number of office-based SB interventions, including the provision of sit-stand workstations, information, prompts, management support, and appointing team leaders, although study quality is often poor with mixed effectiveness for reducing workplace SB [13–15]. Interventions that target numerous levels of influence via multi-components appear to be more effective [13, 14].

Levels of hybrid working increased following COVID-19, allowing employees to work from home (WFH) [16]. For employees who began WFH during the pandemic, systematic reviews show increases in SB, with one citing an increase of 16% [17, 18]. Research examining the introduction of a flexible working policy prior to the pandemic has also reported both an actual, and perceived, increase in occupational SB [19, 20]. Whilst WFH has benefits, the intersection between work and home life will likely result in unique influences on SB, with evidence suggesting interventions for the office may not directly translate to the home, demanding novel approaches [21–24].

Older employees (≥ 50 years) [25, 26] form one third of the UK workforce and may experience different challenges compared to their younger counterparts due to a range of physical, psychological, and social characteristics [26, 27]. Older workers are more likely to be managing numerous health conditions, with ill-health a key reason for leaving work [27, 28]. Moreover, many older workers who leave work early have a desire to return but face barriers relating to health, caring responsibilities, poor workplace flexibility, inadequate support, and age discrimination [27, 29]. Although granular data are limited, many older employees in desk-based roles have the option to WFH, making it important to understand how this setting influences their sitting behaviours [16, 30]. Prolonged SB and the associated health risks are an occupational hazard, and given that employers have a legal duty of care to protect employee health and provide safe working conditions, they have a responsibility to prevent harm and promote good health before retirement, which can be achieved via evidence-based interventions [31].

When developing interventions, efforts should be made to consider the unique workplace context, engage with relevant stakeholders, and identify key uncertainties [32]. The application of behaviour change theories and frameworks will also be necessary to hypothesise why a behaviour does, or does not, occur. The Behaviour Change Wheel (BCW) integrates 19 behaviour change frameworks to support intervention design and evaluation [33]. Its core, the COM-B model, conceptualises behaviour as arising from interactions between Capability, Opportunity, and Motivation, and can be used with the Theoretical Domains Framework (TDF) to analyse complex behaviours [34, 35]. The TDF synthesises constructs from 33 behavioural theories into 14 domains, providing a comprehensive, theory-informed framework to support the development of behaviour change interventions. These 14 domains can be mapped to the COM-B model [34] and include: Knowledge; Skills; Social/Professional Role and Identity; Beliefs about Capabilities; Optimism; Beliefs about Consequences; Reinforcement; Intentions; Goals; Memory, Attention and Decision Processes; Environmental Context and Resources; Social Influences; Emotions; and Behavioural Regulation.

Combining the COM-B and TDF has been used to explain workplace SB and develop tailored interventions in office settings [36–41]. Only one intervention, the multi-component *Stand More AT (SMArT) Work*, has been successfully tested as a large-scale randomised controlled trial, reporting significant reductions in workplace SB [42]. Combining the COM-B, TDF, and BCW to explore SB in the homeworking setting is sparse. Niven et al., [43] used the COM-B to examine the sitting behaviours of homeworking university employees. The sample, on average, spent 89.5% of their time sitting and a range of barriers to interrupting sitting were identified, including work demands, being immersed in work, belief of negative impact on productivity, and a lack of intention to reduce sitting. Unique findings related to the lack of space and equipment in the home, as well as a social pressure to remain seated, suggesting that social norms and expectations relating to sitting behaviours persist via digital communication. This present study extends on the work by Niven by focusing specifically on older employees, an underexamined group, and integrating the COM-B and TDF to provide a comprehensive behavioural analysis of SB in the home environment.

A more in-depth qualitative exploration of influencing factors in the home environment is essential, especially in older employees, to develop suitable interventions [44]. Thus, this study sought to use both the COM-B and TDF to explore how older employees understand and experience SB when WFH, and to answer the research question of ‘what barriers and enablers to interrupting SB exist for older employees when WFH?’.

## Methods and Materials

This qualitative study is the first stage of a project aiming to develop an intervention to reduce and interrupt SB in a group of older employees aged ≥ 50 when WFH in desk-based occupations. The study is reported in line with the COnsolidated Criteria for REporting Qualitative Studies (COREQ), which can be found in Supplementary file 1, including more detail regarding the philosophical position and reflexivity of this work [45].

### Design and Participant Recruitment

This study adopted a qualitative methodology to allow for an in-depth exploration of individual employees’ experiences and to uncover the nuanced multi-level influences on SB in an under-researched setting. To capture each participant’s unique perspective while ensuring confidentiality, one-to-one semi-structured interviews were used rather than focus groups [46]. Semi-structured interviews allow for a flexible conversation, whilst the presence of a structure ensures sufficient data is collected on the topic.

A convenience sample of 22 participants were recruited from a local council workplace in Greater Manchester, most of whom [21] identified as White, which is acknowledged as a limitation. There was no relationship between the researcher and participants before the commencement of the study. A recruitment email was circulated by a member of the employer’s wellbeing steering group and also shared with the employer’s diversity group. Eligibility criteria included: aged ≥ 50 years old, predominantly desk-based job role, work from home at least two days per week (or part-time equivalent), and able to stand up and walk.

### Materials

Participants completed an online Qualtrics questionnaire to collect demographic information: name, email, age, gender identity, ethnic group, education level, living status, current job role, years in job role, hours per week, and average days WFH each week. The interview guide was developed and based on the TDF and included questions adapted from previous work [36, 37]. Questions not relating to the TDF were also included, created with the research team, to ensure a comprehensive interview (Supplementary file 2). The schedule was pilot tested with two older employees who had taken part in previous public involvement work and the research team to address question suitability, identify potential weaknesses, gain interviewing experience, and estimate interview duration [47–49]. Following piloting, the term ‘sedentary behaviour’ was replaced with ‘sitting’ to improve clarity and focus, some questions were reworded to be more open-ended, and two questions with prompts were added to explore barriers and facilitators not previously mentioned.

### Procedure

Participants received a participant information sheet and provided informed consent via a consent form. Before the interview, participants completed a demographic survey via Qualtrics using a link only sent to them. Interviews were conducted virtually within working hours, lasting between 24 and 55 minutes (mean length: 37.7). Only the participant and researcher (LM) were present during interviews, which were audio-recorded and transcribed. Field notes were taken during the interviewing and analysis process to allow for reflection and data immersion. Transcripts were not returned to participants for comment or corrections. Participants received a £15 voucher for successful completion of the interview.

### Analysis

Anonymised transcripts were uploaded to NVivo 12.7.0 (QSR International Pty Ltd) and analysed using reflexive thematic analysis [50]. With this approach, themes do not ‘emerge’ from the data but instead are generated via an active process in which the researcher uses their theoretical lens to produce new knowledge. Data saturation was not used to inform the final sample size of this study as this approach relies on the data being transparent and obvious prior to the construction of themes, which is incompatible with reflexive thematic analysis [51]. Instead, it was anticipated between 15 and 25 interviews would take place with the final sample guided by the concept of information power, which encourages a reflection on the richness of data provided and is recommended by Braun and Clarke for this analysis approach [52].

Coding was conducted by the lead author with regular reviews of the process taking place with a second researcher (AP) and meetings with the wider research team to sense check codes and theme development. The six-step process provided by Braun and Clarke to guide the analytical process is a strength, as it ensures a systematic approach to TA [53, 54]. Initial coding was conducted on a semantic level, capturing the explicitly expressed surface meaning of the data. As the coding process progressed, latent interpretations were used to examine and identify the underlying assumptions, ideas, and conceptualisations of the data where appropriate. The updated six-step process of reflexive TA, and the corresponding actions are outlined in Supplementary file 3.

The data was first analysed inductively to develop themes and sub-themes, followed by deductively mapping themes and sub-themes to the TDF and COM-B model. This approach was informed by previous research on SB both in the workplace and other settings which have successfully adopted this hybrid analysis approach using the TDF and COM-B model [36, 37]. The lead researcher was a White female PhD student in their 20s with a background in public health with prior qualitative research experience and believes this method is well-suited for exploring participants’ experiences. The age difference and role in studying activity behaviours may have influenced how participants were perceived and what they shared. A reflexive journal was used to consider how this background and assumptions may have influenced data collection and interpretation. More details are reported in Supplementary material 1. The wider research team also work in the field of healthy ageing.

## Results

### Participants

Twenty-two interviews were conducted between February and March 2024. Participants were employed across a range of departments and their demographic characteristics are reported in Table 1.

**Table 1:** Participant characteristics

Characteristic	Total (N = 22)
Age	
Mean (*SD*)	56.1 (4.2)
Gender Identity	
Woman	12 (55%)
Man	10 (45%)
Ethnicity	
Asian or Asian British	1 (5%)
Black, Black British, Caribbean or African	0
White	21 (95%)
Mixed or multiple	0
Highest Level of Education	
Left school before taking GCSE	0
GCSE or equivalent	3 (14%)
A-Levels of equivalent	5 (23%)
University degree or higher	14 (64%)
Living Status	
Alone	2 (9%)
With partner	14 (64%)
With family or friends	6 (27%)
Managerial Status	
Manage others	11 (50%)
Do not manage others	11 (50%)
Years in job Role	
Mean (*SD*)	7.8 (7)
Hours worked per week	
Mean (*SD*)	34.3 (5.2)
Reported average number of days/week working from home	
1	0
2	2
3	6
4	8
5	6

### Themes

**Fig. 1:**
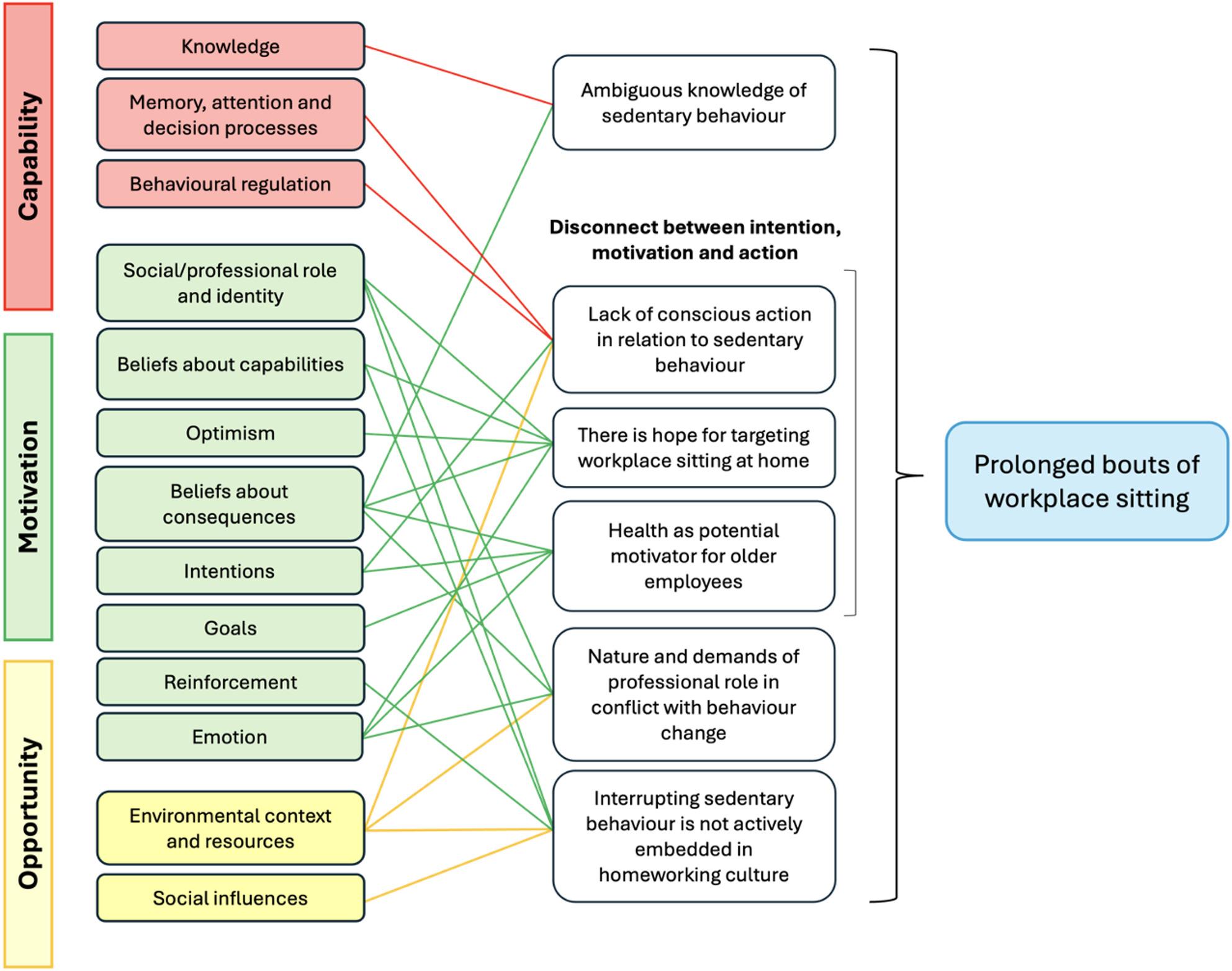
Themes linked to the TDF and COM-B components

The inductive analysis led to the development of six themes (Fig. 1). Themes 3, 4, and 5 are connected under an overarching theme, ‘Disconnect between intention, motivation, and action’. Fig. 1 and Table 2 show the deductive analysis and links to relevant TDF and COM-B domains. Key themes are presented with their assigned COM-B diagnosis and supporting quotes including the participant number, gender, and age.

**Table 2:** Themes linked to TDF and COM-B components

Theme	COM-B component	TDF category
Ambiguous knowledge of sedentary behaviour	Psychological Capability Reflective Motivation	Knowledge Beliefs about consequences
Nature and demand of professional role in conflict with behaviour change	Reflective Motivation Automatic Motivation Physical Opportunity	Social/professional role and identity Beliefs about consequences Emotion Environmental context and resources
Lack of conscious action in relation to sedentary behaviour	Psychological Capability Reflective Motivation Physical Opportunity	Memory, attention and decision processes Behavioural regulation Intentions Environmental context and resources
Health as potential motivator for older employees	Reflective Motivation Automatic Motivation	Beliefs about consequences Intentions Goals Emotion
There is hope for targeting workplace sitting at home	Reflective Motivation Automatic Motivation	Social/professional role and identity Beliefs about capabilities Optimism Beliefs about consequences Emotion
Interrupting SB is not actively embedded in homeworking culture	Reflective Motivation Automatic Motivation Physical Opportunity Social Opportunity	Social/professional role and identity Beliefs about capabilities Reinforcement Environmental context and resources Social influences

#### Theme 1: Ambiguous knowledge of sedentary behaviour (psychological capability and reflective motivation)

Participants often referred to SB as a lack of movement characterised by high levels of sitting, which indicated a somewhat accurate understanding of SB. However, none were able to identify all three defining features of SB (energy expenditure, waking behaviours, and posture). The ambiguity of the phrase ‘not moving’ became clear as conversations progressed and it appeared that SB was frequently misconstrued as the absence of PA, as opposed to it being its own distinct behaviour. Thus, when discussing the need to reduce SB, some participants interpreted this as needing to engage in longer periods of PA, as opposed to short and frequent breaks from sitting: “*I am conscious that I need to move around*,* like for instance*,* I’ll go for a walk every lunchtime” [P10*,* Man*,* 64].* The priority placed on PA was further highlighted by some participants who engaged in PA to *“try and combat the sedentariness of it [sedentary work]” [P15*,* Woman*,* 60].*

Whilst participants appeared to be aware that SB was damaging to health, there was uncertainty: “*I know sitting down for ages isn’t good for one*,* you know*,* I couldn’t really articulate why” [P22*,* Man*,* 59].* Musculoskeletal issues, poor posture, and general aches and pains of the back and lower limbs were frequently mentioned. Weight gain, a negative impact on eyesight, and poor mental wellbeing were also highlighted although few referenced the serious implications for cardiovascular health, suggesting limited knowledge regarding the physiological responses occurring within the body.

There was also uncertainty regarding the health benefits to be had from interrupting SB, although most acknowledged this would be a positive action. For some, the lack of quantifiable national guidelines regarding SB, and its associated health risks, contributed to an apparent view that SB was not distinct from PA: *“if I actually knew how much damage it was doing me*,* I’d probably do more of it [interrupt sitting]” [P4*,* Man*,* 62]*.

#### Theme 2: Nature and demand of professional role in conflict with behaviour change (reflexive motivation, automatic motivation, and physical opportunity)

High levels of workplace SB were viewed as an unavoidable feature of desk-based work: *“if I’m working from home*,* how else can I work if I don’t sit” [P4*,* Man*,* 62*]. Most referenced the demands and pressure of work, which appeared to act as a barrier to interrupting SB. For some, their apparent desire to complete work tasks meant negative emotions such as anxiety, stress, frustration, and guilt, were associated with interrupting SB: *“I’m anxious about getting things done. So*,* it’s always playing on my mind*,* even when I’m away from work*,* away from my desk” [P2*,* Woman*,* 61]*.

The importance of completing work tasks was further highlighted by the common perception that regularly interrupting sitting would negatively impact productivity and concentration, contributing to the belief that changing sitting behaviours may be incompatible with work demands: *“I think I would achieve a lot less…And there is the danger that when you walk away from your desk you’ve got to get back up to speed with whatever you were doing” [P15*,* Woman*,* 60]*. These apparent perceptions held by participants, and their motivation to interrupt SB, were also influenced by the specific demand and nature of their work task: *“I’ve got a task that I’ve got a deadline for at the end of today*,* so…I will have less breaks because I need to have that focus to get it completed” [P21*,* Woman*,* 50].*

Moreover, homeworking resulted in more online meetings which many highlighted as facilitating prolonged sitting. The perceived opportunity to interrupt sitting during an online meeting was dictated by its nature as well as the level of contribution required by the individual, often leaving participants feeling tied to their desk: *“I could*,* actually…get up and wander around in a meeting*,* but you’re sort of making notes…It’s quite difficult to do*,* there’s only a certain type of meeting where you could do that” [P12*,* Woman*,* 50].* Comparisons were also drawn with the office in which meetings would facilitate movement with colleagues, demonstrating how WFH can alter movement behaviours: *“If you had a meeting in the office*,* you have to actually physically get up and walk to that meeting*,* whereas if you have a meeting at home*,* you just sit” [P1*,* Woman*,* 56]*.

### Overarching theme: Disconnect between intention, motivation, and action

This overarching theme encompasses themes 3–5, which are anchored together through the shared concept of a disconnect between the intention, motivation, and action to interrupting prolonged sitting when at home.

#### Theme 3: Lack of conscious action in relation to sedentary behaviour(psychological capability, reflective motivation, and physical opportunity)

For most participants, the interruption of workplace sitting appeared to be triggered by a random event or a specific need, as opposed to a conscious intention to regularly break up prolonged sitting. Interrupting sitting had a purpose, ranging from wanting to ease physical discomfort, needing a comfort break, and completing household tasks: *“The thinking behind [interrupting sitting] isn’t to stop me from sitting down*,* the thinking behind it there’s stuff to do so I do it. But I realise that it’s a good thing to do anyway*,* but that’s not the principal reason for doing it…. I don’t have a conscious approach to any of this” [P22*,* Man*,* 59].* Triggers to move were largely influenced by an individual’s unique interactions with their home environment, often due to the increased availability and frequency of external stimuli: *“if you’ve got reason to get up*,* you get up*,* like the door*,* the doorbell rings*,* a delivery driver and things like that…they’re more unusual events” [P10*,* Man*,* 64].* There was, however, some indication that working in a private and separate room could minimise distractions and thus contribute to becoming more engrossed in one’s work which facilitated prolonged sitting: *“Because I have an office space that I can shut the door on and there aren’t any interruptions*,* I probably have less interruptions that I need to get up and move” [P21*,* Woman*,* 50].* In contrast, some participants stated that working in a more open space at home, where distractions were more readily available, could trigger movement: *“I’m near the kitchen so I can brew up…sometimes you’ll hear the washer finish and you’ll get up for that” [P16*,* Woman*,* 53].*

Considering the lack of intent participants had to interrupt their sitting, and the reliance on external triggers for movement, the subconscious and habitual nature of SB played a critical role. Although participants reportedly had an awareness of how much sitting they engage in, almost all made reference to not realising how much time had passed and forgetting to move, often due to being engrossed in work. There appeared to be a lack of psychological tools for participants to take notice and regulate their SB, further demonstrating the lack of conscious action. Some participants highlighted the usefulness of setting up reminders to move as well as self-monitoring, to become more conscious of their behaviour: *“I can be sitting for two hours and not realise… If they [the employer] could set up this timer that goes off every 20 minutes that would be really helpful.” [P9*,* Woman*,* 60]*.

#### Theme 4: Health as potential motivator for older employees (reflexive motivation and automatic motivation)

Health appeared to be central to the identity of participants as older employees. Most expressed a heightened awareness of their changing health as a result of ageing, which ranged from increased MSK aches and pains, the onset of menopause, and deteriorating eyesight. Almost all made reference to experiencing physical discomfort at work, with some believing their symptoms were exacerbated by excessive SB: *“I’m at that stage of life where you get more aches and pains anyway*,* but I don’t think sitting down has helped” [P3*,* Man*,* 52]*. Participants shared a desire to protect their health and there was recognition that interrupting sitting was something participants should, and need, to do as older employees. Concerns about how this could be managed alongside work troubled some participants: *“I don’t like to get up and move about much because I feel as though I’m skiving*,* so*,* and then I think as an older employee I know I need to do that” [P16*,* Woman*,* 53].* Despite participants sharing an awareness of how important it would be to change their sitting behaviours, this did not necessarily translate to action: *“And every time I feel a crink*,* you know*,* a twinge in my back*,* it’s always*,* ooh*,* I need to do more. And do I? No*,* I don’t” [P8*,* Woman*,* 56]*. Moreover, two participants shared how WFH helped them manage their health conditions which allowed them to remain in the workforce.

#### Theme 5: There is hope for targeting workplace sitting at home (reflexive motivation and automatic motivation)

The central concept of this theme relates to the positive feelings, desires, and expectations held by participants in relation to interrupting workplace sitting. Participants reported positive feelings when interrupting their sitting such as: *“better” [P1*,* Woman*,* 56]*,* “refreshed” [P16*,* Woman*,* 53]*, and *“energised” [P21*,* Woman*,* 50].* In contrast, prolonged bouts of SB triggered negative emotional responses: *“sluggish” [P11*,* Man*,* 50]*,* “lethargic” [P3*,* Man*,* 52] and “irritated” [P9*,* Woman*,* 60]*. Some acknowledged these negative feelings were driven by their workload, the screen-based nature of the job, as well as the lack of social and physical interaction with colleagues when WFH. There was a desire to experience more of the positive feelings associated with interrupting SB and simultaneously reduce the negative effects of prolonged SB. Frequently cited was the expectation that interrupting SB would offer a mental break from work, which some anticipated would have a positive influence on their productivity and concentration: “*it [interrupting sitting] would make me work better*,* I’m sure*,* and feel better and more productive” [P13*,* Woman*,* 52].* Participants also anticipated that interrupting sitting would relieve discomfort and improve their psychological health: *“If you’ve got to have a walkabout*,* you’re not feeling as tired and as stiff as you were…maybe that improves your concentration and improves your mental health as well as your physical health.” [P6*,* Man*,* 56]*.

Most reported that it was realistic to regularly interrupt their sitting every 20 to 30 minutes although there were some concerns regarding how this could be managed with their work. For some, interrupting SB every hour was reportedly more realistic than at 20-minute intervals due to their workload and desire to remain focused.

A key factor in the hope for targeting workplace sitting related to the level of perceived behavioural control, which participants largely reported as being high. For some, working in their home appeared to contribute to this sense of control as the working culture and social norms associated with sitting differed to when in the office. For example, some shared they could interrupt their sitting when at home as there was no one *‘keeping an eye’ [P9*,* Woman*,* 60]* on their behaviours: *“I feel I can get up when I need to get up or when I want to get up*,* rather than when I’m in the office I probably won’t get up*,* because in the office there’s a more formal culture” [P14*,* Man*,* 55]*. Feelings of trust between employees and their team, as well as the flexible working policy in place, were also emphasised as reasons for feeling in control.

These high levels of perceived behavioural autonomy appeared to contribute to participants feeling confident they could alter their sitting behaviour in the future. Interestingly, some participants believed this confidence was related to their position as an older employee due to having more workplace experience as well as changing priorities in relation to health: *“I’m 50 years old*,* I’ve been doing my job for a long time… I’m not going to get sacked for getting up and walking round for five minutes.” [P11*,* Man*,* 50]*.

#### Theme 6: Interrupting SB is not actively embedded in homeworking culture (reflective motivation, automatic motivation, physical opportunity, and social opportunity)

Participants discussed a perceived poor culture surrounding back-to-back and lengthy online meetings which facilitated prolonged sitting: *“I think there’s a culture of just blocking up people’s time” [P13*,* Woman*,* 52].* Social norms appeared to influence sitting behaviours during online meetings with many acknowledging that interrupting sitting was not something they felt able or comfortable to do. For some, this was influenced by the perception that regularly interrupting sitting was associated with not working and thus was incompatible with their professional identity. Social norms were further influenced by who was on a call, as well as the individual’s required contribution to the meeting, demonstrating the perceived existence of sub-cultures between and within teams: *“If there are about ten people on a call or fewer*,* I will stay sitting throughout. If there are more than ten*,* I’ll turn my camera off and I’ll wander off and do things…social convention means that I feel I ought to stay looking as if I’m paying attention.” [P19*,* Man*,* 56]*.

Almost all made reference to their employer promoting workplace wellbeing, although some felt this was *“more lip service” [P2*,* Woman*,* 61]* that was not actively reinforced or compatible with the demands of the job: “*with culture you say one thing*,* and expect one thing*,* but actually the reality of the job is totally different” [P12*,* Woman*,* 50].* There appeared to be some workplace initiatives in place but these targeted PA and were predominantly for office-based employees. No participants reported an awareness of workplace policies or initiatives specifically for SB when WFH. When discussing the lack of workplace policies, one participant expressed *“those of us that work at home more regularly have been a bit left at home”* and there could “*be more emphasis on what we could be doing at home” [P3*,* Man*,* 52]* with regards to SB.

The shift to homeworking prompted discussions regarding where the responsibility lay for interrupting workplace SB. Many viewed their sitting behaviours as an individual responsibility which required high levels of internal motivation. For some, this was influenced by the belief that the influential role of their employer was diminished in the homeworking environment, exacerbated by colleagues and teams working in silos from one another, making group approaches to interrupting SB more challenging: “*it’s definitely more personal. I’m not sure the microbreak thing*,* given that we’re all working remotely in different places*,* how a group thing will work” [P6*,* Man*,* 56].* Despite these views, social norms appeared to influence sitting behaviours, albeit these manifested themselves in a different way to the office. Participants shared how colleagues, and their approach to online meetings, could influence sitting behaviours and increased conversations and encouragement to interrupt sitting would be beneficial. In addition, there was a desire for managers to actively promote and reinforce employees to interrupt their sitting, which would be easier if formal policies and initiatives were developed. Some highlighted these policies could target online meetings to ensure they allowed for breaks. Some also noted it was important to ensure the content and delivery of policies respect individual autonomy.

For some, social norms in the homeworking environment manifested themselves as a perceived lack of trust, which contributed to an expectation and pressure to be at their desk: “[I] *sometimes feeling a bit guilty about*,* I’m going to take a bit of a break now but I’m probably not really allowed to so I need to do this really quickly” [P7*,* Woman*,* 55].* Reportedly, this appeared to be exacerbated by the lack of reinforcement and policies in place to support the interruption of sitting, further demonstrating the complexity of colleagues working in silos from one another and the existence of sub-cultures when WFH: *“there’s something about cultures of trust… some teams are more trusting than other teams…that some managers just don’t know how to trust their employees to just get on with it” [P19*,* Man*,* 56].*

## Discussion

### Ambiguous knowledge of sedentary behaviour

The apparent lack of understanding of SB and the associated health implications reported in this study is similarly highlighted in previous work [37, 38, 55, 56]. Not knowing the frequency and the type of activity that should take place when interrupting SB has also been identified as a barrier in other workplace populations [36, 55]. Moreover, as reported in previous work, some participants held the belief that PA could compensate for workplace SB [43, 56]. Evidence indicates that the health risks associated with SB are independent of PA [3] and whilst some studies suggests PA can have attenuative or eliminative effects, this requires high levels of activity [57]. This is perhaps unlikely to be achieved considering the habitual nature of SB which often displaces time for PA [58, 59]. Thus, employees should avoid compensatory behaviours and instead reduce both physical inactivity and SB, by targeting psychological capability and increasing knowledge about SB. This study builds on existing work by highlighting that older employees may also have limited knowledge of SB and its health impacts, underscoring the need to address these misconceptions in workplace interventions. These findings add value, as they contrast with those reported by Niven et al., [43] and indicate that a lack of knowledge about SB may also exist in homeworking populations, addressing Niven’s suggestion to explore if education-focused interventions are necessary in this setting.

### Nature and demand of professional role in conflict with behaviour change

High workload, time pressure, and task demands may contribute to feeling tied to one’s desk, highlighting the potential tension between completing tasks and movement [36, 37, 43, 55, 60–63]. In office populations, sit-stand workstations are often used and have been shown not to decrease worker productivity [64, 65] but may even enhance productivity, compared to those who remain seated [66]. However, their use may not be transferable to the home environment due to practicality and affordability issues [23]. Whilst there is no standard measure of work productivity, and various measures were used, one review indicated that active microbreaks of 2–3 minutes every 30 minutes may be more achievable for homeworkers without negatively affecting productivity [67]. Challenging the existing perceptions relating to the consequences of interrupting SB when WFH will be important for encouraging behaviour change. Research on low-cost interventions and how these impact productivity would be beneficial.

Participants believed that the shift to virtual communication resulted in prolonged sitting, with comparisons drawn to the office environment, where connection and collaboration would facilitate movement. Similar beliefs were reported in a study prior to the pandemic, in which home-based employees reported an increase in SB, with some attributing this to the rise of online meetings and the lack of unplanned interactions with colleagues at the office [19]. The visibility of colleagues and regular face-to-face interactions has been associated with shorter bouts of sitting in office environments, components lost when WFH [68, 69]. The current study supports work conducted during the pandemic which speculated that increases in SB for those WFH could be due to increased virtual communication [70]. This virtual communication also takes place in the office, given the hybrid nature of work, and thus employers should consider how online communication is managed to discourage prolonged SB [71].

### Lack of conscious action in relation to sedentary behaviour

Despite participants expressing a conscious awareness of their high levels of workplace sitting, this did not translate to action. The sitting behaviours of participants were largely subconscious and a result of automatic habitual processes, consistent with previous work [36, 37, 39, 43]. It has been suggested that the act of sitting may not be a meaningful action, and is instead a by-product of engaging with more important activities, such as work [72]. This may contribute to SB as a subconscious habit and thus raising awareness of one’s sitting is likely to be a critical antecedent to changing behaviour. Making plans to change behaviour and to engage with an intervention may also be useful for homeworkers [61].

Reliance on external cues and the variability of the home environment may lead to irregular interruptions of sitting, potentially resulting in prolonged sedentary bouts, a pattern also observed in office spatial design [68, 69]. There may be additional complexity associated with designing and implementing interventions for homeworkers, in which the employer no longer has the same control or influence over the physical surroundings [12]. Future interventions should be mindful of this and focus on low-cost intervention options that target purposive movement and increasing employee consciousness of their sitting behaviours. This is a useful contribution to the literature and highlights the important role of the environmental context when WFH, which warrants future research.

### There is hope for targeting workplace sitting at home

Emotional influences can be important intrinsic drivers for influencing SB, as highlighted in this work and previous [36]. When considering the consequences of changing their sitting, most participants anticipated positive outcomes which were typically associated with increased productivity and concentration, contradicting some of the concerns in Theme 2. Various behavioural theories recognise the importance of individuals holding positive beliefs about outcomes of engaging in a behaviour as a key motivational antecedent to change [73]. Thus, linking the automatic emotional responses to these more reflective beliefs could be targeted in an intervention to foster motivation.

Most reported high levels of perceived behavioural control in terms of their workplace sitting which, for some, appeared to be closely connected to WFH and the perception of a supportive working culture, demonstrating the complex merge of undertaking work in one’s own home and its potential impact on behavioural autonomy at work. The Theory of Planned Behaviour argues that perceived behavioural control can increase one’s motivation and intention to take action [73]. However, the perceived control in this current study did not translate to conscious intentions to interrupt SB, demonstrating the need to consider wider determinants of workplace SB, especially those relating to unconscious thought-processes. Some participants believed their position as an older employee resulted in increased confidence to change their sitting behaviour. Although positive stereotypes do exist [74], older worker identity is more often associated with age discrimination, negative attitudes, and stereotypes [74]. Thus, exploring the ways in which identity manifests in the homeworking environment, and how this influences SB, could be explored further.

### Health as potential motivator for older employees

The topic of health appeared to be central to the identity of participants, with most acknowledging the presence of health conditions, a key reason for older workers exiting employment [27–29]. Participants acknowledged the potential health implications associated with ageing and the challenge of managing this alongside work before retirement. Many reported the sedentary nature of their job contributed to physical discomfort and shared that changing their behaviour was something they needed to act on. The use of words such as ‘need’ and ‘should’ in relation to interrupting workplace SB indicated an apparent lack of intrinsic motivation for some participants. Thus, despite an apparent awareness that sitting less could protect their health, it was not necessarily something they intended or wanted to do, highlighting the disconnect between their intention, motivation, and action. The value placed on health has been identified in previous work and the findings of the current study indicate this may be heightened for older employees experiencing health challenges [55]. Health could be used to target motivation and address some of the ambiguous knowledge relating to SB. Finally, although only referenced by two participants, the home environment helped with the management of health conditions, which warrants further exploration considering the ageing workforce and the need for more flexible working options to support economic activity [26, 27].

### Interrupting sedentary behaviour not actively embedded in homeworking culture

Organisational culture influences the values, social norms, policies, and belief systems of a workplace, and has been identified as a barrier to interrupting sitting in office settings [75, 76]. This study demonstrated how social norms relating to sitting continue to manifest at home, particularly during online meetings, where participants perceived an expectation to remain seated even without physical proximity to colleagues. This is consistent with Niven et al., [43] who also identified pressures to remain seated when WFH. Our findings extend this work by highlighting that these norms remain influential for older employees and are reinforced in part by the digital work environment. These results highlight that social norms persist across different settings and populations, suggesting future interventions foster a social environment, both in physical and digital workspace, that encourages the interruption of sitting into employees’ professional identity.

The current study also highlighted the need for employees to take a personal responsibility for their sitting whilst recognising the importance of encouragement and reinforcement from the employer, which could be critical to the success of SB interventions [36, 55, 62, 63]. The apparent lack of policies targeting SB in this study could be influenced by the lack of quantifiable national guidelines, making employers wary of issuing advice, although future research should explore this.

Some participants noted that interventions should ensure the content and delivery of any policies or initiatives respect autonomy [77]. The role of trust between employees and the employer was highlighted by some participants who shared a perceived expectation to remain at their desk when WFH, similarly reported for office-based employees [37, 60]. Organisational strategies have formed part of larger multi-component interventions to target SB, and although the contribution of these components is poorly understood, they may be essential for embedding trust and empowering individuals to interrupt their sitting [14].

### Strengths and Limitations

A strength of this study is the use of the COM-B model and TDF, which allow for a systematic behavioural diagnosis of SB, facilitating the future development of interventions targeting these specific influences. This study addresses a gap in the literature by focusing on older employees and contributes to a growing body of work exploring the homeworking environment and its influence on SB. This work offers an insight into the target population and setting context, which will be critical for the development of homeworking interventions.

Despite a useful split in terms of gender identity and range of ages in the sample, there was poor ethnic diversity, with most identifying as White. Thus, the insights in this work may overlook the homeworking experience of other ethnic groups, which is important in the design of suitable workplace interventions, especially given the different health and work inequalities ethnic minorities experience [16, 25, 27]. In addition, participants were recruited from one public sector employer which operated a flexible working policy. As a result, the experiences of older employees in other employers, such as the private sector with more inflexible working arrangements, are not reflected and different influences may be at play. Although the themes identified in this study could be transferable to similar populations of homeworkers, the influence of individual organisational culture must be considered.

## Conclusions

This study addresses a gap in the literature by considering how older workers who WFH experience SB, using a rigorous and theory-driven methodology. A range of factors were reported and mapped across the COM-B and TDF, indicating the complexity of workplace SB. Reflective motivation will likely be critical in changing behaviour with all themes linking to this component. Moreover, the most referenced TDF domains (Social/professional role and identity; Beliefs about consequences; Emotion; and Environmental context and resources) all explore the impact changing SB may have on one’s identity as an employee when WFH, demonstrating the importance of balancing an intervention with the roles and responsibilities of employment, and the influence of organisational culture.

Participants highlighted some known barriers, specifically those relating to ambiguous knowledge, organisational culture, the habitual nature of SB, the priority placed on work tasks, and the role of the physical environment. A key contribution of this paper is that the home environment is a major influencing factor on workplace sitting, opening up possible avenues for future research.

These findings have practical implications for intervention design, emphasising the need for low-cost and feasible strategies such as increasing knowledge, enhancing management support, and using digitally delivered prompts. In terms of policy, organisations could consider implementing flexible meeting policies and encouraging regular breaks to create a supportive homeworking environment that facilitates the interruption of prolonged sitting. In addition, health appears to be important for older workers and can be used to target motivation when developing suitable interventions in this group. Based on the findings of this study, there is a need to develop low-cost interventions that could be feasibly delivered across a range of home and hybrid settings. Whilst employees of all age groups will likely benefit from such an intervention, it may be particularly useful for older workers who may experience health challenges. Given the rise of homeworking, the dangers associated with prolonged sitting, and our ageing workforce, this study is an important contribution to the literature. Subsequent work would benefit from utilising the BCW and COM-B model to identify appropriate intervention functions, policy options, and behaviour change techniques in a systematic and theory-driven approach.

## Supplementary Information

Supplementary Material 1.

Supplementary Material 2.

Supplementary Material 3.

## Data Availability

Data available upon request. Contact the lead author lily.mott@manchester.ac.uk.

## Abbreviations

BCW: Behaviour change wheel
COM-B: Capability, opportunity, motivation – behaviour
PA: Physical activity
SB: Sedentary behaviour
TDF: Theoretical domains framework
WFH: Working from home
