# Supplementary material for: Interrupting sedentary behaviour when working from home: a qualitative exploration of older desk-based employees
Source: BMC Public Health. 2026 Feb 19;26:796. doi: 10.1186/s12889-026-26719-4 (PMC12961833; doi:10.1186/s12889-026-26719-4)
Supplement: Supplementary file 1 — Supplementary Material 1. [file 12889_2026_26719_MOESM1_ESM.pdf]

Supplementary file 1: Interrupting Sedentary Behaviour when Working from Home: A Qualitative Exploration of Older Desk-Based Employees COREQ checklist

**Topic**	**Item No.**	**Guide Questions/Description**	**Comments**	**Page number (if applicable)**
**Domain 1: Research team and reflexivity**				
Personal characteristics				
Interviewer/facilitator	**1**	Which author/s conducted the interview or focus group?	LM conducted the interviews	**3**
Credentials	**2**	What were the researcher's credentials? E.g. PhD, MD	LM is a PhD candidate with a Masters in Public Health. The researcher has received training on qualitative methods during the PhD programme.	**3**
Occupation	**3**	What was their occupation at the time of the study?	PhD student	**N/A**
Gender	**4**	Was the researcher male or female?	Female	**N/A**
Experience and training	**5**	What experience or training did the researcher have?	The researcher (LM) was working towards a PhD in public health and healthy ageing. LM has completed training in conducting qualitative research and has previously completed a qualitative research study for their master's dissertation. LM believes qualitative research is the most suitable method for collecting data on the lives and experience of people, and that these are shaped by the participant's subjective construction of reality. The researcher had to manage	**N/A**

			numerous assumptions about SB in the homeworking environment and what factors play a role.	
Additional information			The researcher (LM) is a White British female aged in their late 20s. The age difference in interviews, due to the target population being aged ≥50, may have influenced how the researcher is perceived and consequently what information participants chose to share. The researcher had no prior relationship to any of the participants taking part in the study which required additional time and effort to build trust and rapport. Power relations were present during the recruitment stage as the employer circulated the research opportunity to employees. The researcher was aware that employees may have felt pressured to take part and it was made clear that this was an optional study with no personal or professional consequences for not being involved. Although it was made clear all data was anonymous, the researcher was aware that the participants role as an employee may have influenced the information shared regarding their experience when working from home. The position of the researcher as a PhD student researching activity behaviours may also influence what was shared by participants, through fear of being judged.	**N/A**
Relationship with participants				
Relationship established	**6**	Was a relationship established prior to study commencement?	No relationship prior to study	**3**
Participant knowledge of the interviewer	**7**	What did the participants know about the researcher? e.g. personal goals, reasons for doing the research	Information about the PhD research was detailed in recruitment emails and the participant information sheet	**3**
Interview	**8**	What characteristics were reported about	At the start of the interviews the researcher provided information about the research topic and that the long term goal was to develop an intervention	**N/A**

characteristics		the interviewer/facilitator? E.g. bias, assumptions, reasons and interests in the research topic		
**Domain 2: Study Design**				
Theoretical framework				
Methodological orientation and Theory	**9**	What methodological orientation was stated to underpin the study? e.g. grounded theory, discourse analysis, ethnography	The behaviour change wheel and theoretical domains framework were used to underpin the research and reflexive thematic analysis was used to map themes to these frameworks. This study was underpinned by critical realism, a philosophical approach combining elements of ontological realism and epistemological relativism which seeks to understand and explain various social events or phenomena. Critical realism argues that whilst a singular reality may exist, this cannot be fully accessed and thus our understanding of the world is constructed through individual subjective perspectives and experiences, which are both complex and multi-layered. This paradigm allows researchers to develop practical recommendations for change in response to the identified mechanisms. With the aim of this work being to develop a rich understanding of the target behaviour and subsequently develop a suitable intervention, this philosophical orientation was deemed useful.	**2, 3,**
Participant selection				
Sampling	**10**	How were participants selected? e.g. purposive, convenience, consecutive, snowball	A convenience sampling approach was adopted	**3**

Method of approach	**11**	How were participants approached? e.g. face-to-face, telephone, mail, email	A member of staff at the employer circulated an email to staff with the opportunity to take part	**3**
Sample Size	**12**	How many participants were in the study?	22 older employees in a predominantly desk-based job who regularly worked from home	**3, 4**
Non-participation	**13**	How many people refused to participate or dropped out? Reasons?	One employee who completed the demographic survey and returned a consent form dropped out before the interview due to technical difficulties	**N/A**
Setting				
Setting of data collection	**14**	Where was the data collected? e.g. home, clinic, workplace	Interviews all took place online via Zoom. Employees were typically based in their home although some were based in the office when they took part in the interview. All interviews took place during the employee's working hours	**3**
Presence of non-participants	**15**	Was anyone else present besides the participants and researchers?	Only the researcher and participant were present on the Zoom call	**3**
Description of sample	**16**	What are the important characteristics of the sample? e.g. demographic data, date	Demographic information of participants is provided in Table 2 of the manuscript. The demographic information covers age; gender identity; ethnicity; education; living status; managerial status; years in role; hours worked per week; and days per week working from home	**4**
Data collection				

Interview guide	**17**	Were questions, prompts, guides provided by the authors? Was it pilot tested?	The interview schedule is provided as an additional file. The schedule was piloted and appropriately amended with two members of a public involvement group prior to the study.	**3**
Repeat interviews	**18**	Were repeat inter views carried out? If yes, how many?	No repeat interviews were conducted	**N/A**
Audio/visual recording	**19**	Did the research use audio or visual recording to collect the data?	The interviews were audio-recorded only	**3**
Field Notes	**20**	Were field notes made during and/or after the interview or focus group?	Field notes were taken at each step of the analysis process, as well as during the interview stage to allow data immersion and reflection	**4**
Duration	**21**	What was the duration of the interviews or focus group?	Interviews ranged from 24 to 55 minutes with an average length of 37.7 minutes	**3**
Data saturation	**22**	Was data saturation discussed?	A discussion of data saturation is included in the manuscript	**3**
Transcripts returned	**23**	Were transcripts returned to participants for comment and/or correction	Time and resource limitations meant transcripts were not returned to participants	**N/A**
**Domain 3: analysis and findings**				
Data analysis				

Number of data coders	**24**	How many data coders coded the data?	All data was coded by LM, as per reflexive TA which relies on the subjective interpretation of the researcher. Regular discussions took place with AP and the wider supervisory team to sense check coding and the development of themes	**3**
Description of the coding tree	**25**	Did authors provide a description of the coding tree?	Figure 1 in the manuscript shows the themes and how they link to the theoretical framework	**5**
Derivation of themes	**26**	Were themes identified in advance or derived from the data?	A hybrid analysis approach was conducted with themes first being inductively developed and then being deductively linked back to the theoretical frameworks (BCW and TDF). The deductive analysis to the TDF and COM-B will support with the identification of appropriate intervention functions to support progression along the BCW phases.	**3**
Software	**27**	What software, if applicable, was used to manage the data?	Nvivo 12.7.0 (QSR International Pty Ltd) was used to manage the transcript data.	**3**
Participant checking	**28**	Did participants provide feedback on the findings?	Due to time and resource limitations the participants did not provide feedback on the findings during this study.	**N/A**
Reporting				
Quotations presented	**29**	Were participant quotations presented to illustrate the themes/findings? Was each quotation identified? e.g. participant number	Quotations were used to support themes and participant numbers were used	**4**
Data and findings consistent	**30**	Was there consistency between the data presented and the findings?	Inter-rater agreement approaches were not used in this work due to their incompatibility with reflexive TA as detailed above	**3**

Clarity of major themes	**31**	Were major themes clearly presented in the findings?	Themes were presented in the results section and supported by a visual image (figure 1)	**4**
Clarity of minor themes	**32**	Is there a description of diverse cases or discussion of minor themes?	The results and discussion show how the major themes link back to the constructs of both the COM-B model and TDF	Results and discussion

Developed from Dossett LA, Kaji AH, Cochran A. SRQR and COREQ Reporting Guidelines for Qualitative Studies. JAMA Surgery. 2021;156(9):875-6 and Tong A, Sainsbury P, Craig J. Consolidated criteria for reporting qualitative research (COREQ): a 32-item checklist for interviews and focus groups. International Journal for Quality in Health Care. 2007;19(6):349-57.
