# Supplementary material for: Interrupting sedentary behaviour when working from home: a qualitative exploration of older desk-based employees
Source: BMC Public Health. 2026 Feb 19;26:796. doi: 10.1186/s12889-026-26719-4 (PMC12961833; doi:10.1186/s12889-026-26719-4)
Supplement: Supplementary file 2 — Supplementary Material 2. [file 12889_2026_26719_MOESM2_ESM.docx]

# Supplementary File 2 for Interrupting Sedentary Behaviour when Working from Home: A Qualitative Exploration of Older Desk-Based Employees

Table shows questions used in interviews and their related TDF domain/reference. Where included, references refer to questions adapted from previous qualitative work.

| **Questions/Prompts** | **TDF Domain** |
| --- | --- |
| Could you tell me a bit about yourself and your job role? |  |
| Has working from home always been the case in your current job role? |  |
| What is your understanding of sedentary behaviour?  How would you describe workplace sedentary behaviour to others? (Brierley et al., 2021) | Psychological capability: Knowledge |
| What do you think the consequences are of sitting for long periods? (Ojo et al., 2019)  *Prompt: Are you aware of the health benefits of being less sedentary?* |  |
| Can you describe a typical day when you work from home?  *Prompt: Can you describe what aspects of your working day are sedentary?* |  |
| When you are seated, how often is it for longer than 20 minutes at a time? (Brierley et al., 2021)  *Prompt: Never, sometimes, most of the time, always* |  |
| Is regularly interrupting your sitting by taking microbreaks something you usually do when working from home?  *Prompt: Why is that?*  Is interrupting your sitting with microbreaks something you want to do more of?  Do you have any current strategies to help you interrupt your sitting with microbreaks? | Psychological capability: Memory, attention and decision processes  Goals/Intentions  Behavioural regulation |
| When you do get up from sitting, how long do you usually spend away from your desk standing or moving? (Brierley et al., 2021) |  |
| What do you think stops you from taking regular microbreaks to interrupt sitting when you work from home?  Prompts:  What role do online meetings play?  *What role does your workload or work tasks play?*  *Does the layout of your home or workspace play a role?* | Physical Opportunity: Environmental context and resources  Social Opportunity: Social influences |
| Is there anything that helps you to interrupt your sitting and take microbreaks when working from home?  *Prompts:*  *Breaks in between meetings*  *Household tasks?*  *Layout of your desk space?*  *Personal motivation?* |  |
| As well as (refer to what they’ve mentioned), are there any other barriers or facilitators in your home environment that influence your levels of sitting when you’re working? |  |
| Can you discuss how your levels of sitting differ between your days in the office and the days you work from home?  *Prompt:*   - *Do the number of microbreaks differ between office and home working?* - *What factors contribute to this?* |  |
| In what ways do your work colleagues influence your sitting time when working from home?  In what ways do your family or friends, or those you live with influence your sitting when working from home? | Social Opportunity: Social influences |
| How does the culture of your workplace impact your levels of sitting and taking regular microbreaks?  *Prompt: Why do you think that is?*  Can you discuss any current initiatives or policies to help you interrupt your sitting and take microbreaks when working at home? | Physical Opportunity: Environmental context and resources  Automatic motivation: Reinforcement |
| When you spend a lot of time sitting when you work from home, how does that make you feel? | Automatic motivation: Emotion |
| How does your mood influence your levels of sitting when working from home? (Ojo et al., 2019) |  |
| And when you do interrupt your sitting, how does that make you feel? |  |
| When working from home, how motivated do you feel to interrupt your sitting with microbreaks?    *Prompt: why is that?* | Reflective motivation: Intentions/Goals |
| How confident are you that you could take more microbreaks when working from home in the future? (Ojo et al., 2019)  *Prompt: Why is that?* | Reflective motivation: Social/Professional role and identity/Beliefs about capabilities/Optimism |
| When working from home, how realistic do you find breaking up long periods of uninterrupted sitting for example a minute or 2 every 20 or 30 minutes  *Prompt: Why is that?* |  |
| What strategies do you think would help you have more microbreaks to interrupt your sitting when working from home?  *Prompts:*  *Online meetings*  *Education*  *Policies*  *Reminders*  *Competitions*  *Any other strategies?* | Psychological Capabilities: Behavioural regulation |
| How do you think your work colleagues or employer help you have regular microbreaks to interrupt your sitting when working from home? (Ojo et al., 2019) | Social Opportunity: Social influences |
| What do you think the consequences would be of having regular microbreaks when working from home? These consequences may be good or bad  *Prompt:*  *Would there be any impact on your health or wellbeing?*  *Would there be any impact on your productivity or workload?(Brierley et al., 2021)* | Reflective motivation: Beliefs about consequences |
| As you are aware, this research is exploring the experience of sitting in older employees who work from home. Is there anything important you would like to add about how being an older employee may influence your experience of what we have discussed today?  *Prompt: Does the health of older employees play a role?* |  |
| How does being an older employee impact the autonomy you feel over your workplace sitting? |  |
| Finally, are there any other important issues or anything you would like to add that we haven’t discussed? |  |

Brierley, M. L., Smith, L. R., Bailey, D. P., Every, S. A., Staines, T. A., & Chater, A. M. (2021). Perceived influences on reducing prolonged sitting in police staff: a qualitative investigation using the Theoretical Domains Framework and COM-B model [Report]. *BMC Public Health*, *21*, NA. <https://link-gale-com.manchester.idm.oclc.org/apps/doc/A686472906/AONE?u=jrycal5&sid=bookmark-AONE&xid=9c8211df>

Ojo, S. O., Bailey, D. P., Hewson, D. J., & Chater, A. M. (2019). Perceived Barriers and Facilitators to Breaking Up Sitting Time among Desk-Based Office Workers: A Qualitative Investigation Using the TDF and COM-B. *Int J Environ Res Public Health*, *16*(16), 2903. <https://doi.org/https://doi.org/10.3390/ijerph16162903>
