# Supplementary material for: Interrupting sedentary behaviour when working from home: a qualitative exploration of older desk-based employees
Source: BMC Public Health. 2026 Feb 19;26:796. doi: 10.1186/s12889-026-26719-4 (PMC12961833; doi:10.1186/s12889-026-26719-4)
Supplement: Supplementary file 3 — Supplementary Material 3. [file 12889_2026_26719_MOESM3_ESM.docx]

# Supplementary file 3 - Reflexive thematic analysis stages and steps undertaken in the analysis process (Braun and Clarke, 2021a; Braun and Clarke, 2022)

| **Reflexive TA Stage** | **Action Undertaken** |
| --- | --- |
| 1. Data familiarisation and writing familiarisation notes | - Reflective diary on initial thoughts during interview process - Data immersion through reading transcripts and adding annotations in NVivo to provoke critical engagement with dataset - Created NVivo mind maps on early thoughts regarding codes |
| 1. Systematic data coding | - Read through each transcript and coded aspects that appear potentially interesting, relevant or meaningful concepts in relation to the study aims. This process was completed a second time to ensure a thorough and rigorous process - A meeting with a member of the supervisory team took place to sense check initial coding ideas and possible assumptions of the data. - Descriptions of each code were added to reflect their meaning |
| 1. Generating initial themes from coded and collated data | - Codes were printed to allow for a more physical and visual approach to grouping initial themes - Meeting with the supervisory team took place to sense check theme development and alternative interpretations. - The meaning of the data evolved throughout this process which was reflected in the re-naming and re-structuring of codes where appropriate. |
| 1. Developing and reviewing themes | - Themes were reviewed against coded extracts with consideration for the following aspects: the central organising concept, theme boundaries and the theme’s specific and unique contribution - Careful consideration was made to ensure each theme offered a conceptual story, instead of a descriptive topic summary - Read through all transcripts to reconnect with the wider context, ensuring themes accurately reflected the entire dataset in relation to the research questions - Meeting with supervisory team to review themes |
| 1. Refining, defining and naming themes | - Initial theme names and definitions were constructed - Final revisions were made following discussions with the supervisory team |
| 1. Writing the report | *N/A* |
